# Digital platforms’ growth strategies and the rise of super apps

**DOI:** 10.1016/j.heliyon.2024.e25856

**Published:** 2024-02-10

**Authors:** Marc Hasselwander

**Affiliations:** German Aerospace Center (DLR), Institute of Transport Research, 12489, Berlin, Germany

**Keywords:** Super apps, Multi-platforms, Growth strategy, Platform envelopment, Diversification, Vertical integration

## Abstract

Super apps allow users to access messaging, payments, e-commerce, deliveries, ridesharing, and many other services within the same app. While there are some very successful and dominant super apps in Asia such as *WeChat*, *KakaoTalk*, *Alipay*, or *Grab*, others, including Elon Musk with *X* (*Twitter*), are aiming to establish super apps in the U.S. and Europe. This explanatory study analyzes the super app phenomenon from a firm-level perspective. It provides preliminary insights on how digital platforms are reaching the super app status, and are evolving from single-purpose to multi-purpose apps. Using data from 380 platforms in the mobility sector, a regression model is estimated to understand which platforms are capable of pursuing a super app strategy: young, agile, and risk-taking firms. I also discuss the case of *Uber* to illustrate the motivations and the various growth strategies that are incrementally paving the way to becoming a super app. Finally, testable propositions and a conceptual model are forwarded to stimulate future research on this timely topic.

## 1 Introduction

Super apps (also written as super-apps or superapps) are a new phenomenon in the realm of the sharing economy. Unlike the concept of an app for a specific service (e.g., online shopping, food delivery, ridesharing, etc.), super apps are all-in-one solutions that offer a full range of personalized services. Roa et al. [1] defines super apps as “mobile applications that in the same environment seek to satisfy different daily needs of consumers without requiring them to download another application.” Note that the term “super” in this context therefore refers to the app's comprehensive offering of services rather than its quality or dominance [2].

From a business model perspective, super apps can be seen as a distinct category of digital platforms (also referred to as marketplaces or transaction platforms) (cf. [3]). Super apps capitalize on smartphones to facilitate connections between various user groups (e.g., seller and buyer, driver and passenger, etc.) for multiple physical/digital products as well as online/offline services in the same app, thus creating a set of integrated platforms (so-called multi-platforms or platform conglomerates) [4].

Especially in Asia, initial social media and communication platforms (*WeChat*, *LINE*, *KakaoTalk*) and e-commerce platforms (*Alibaba*, *Shopee*) evolved from a single-purpose app to a multi-purpose super app [2]. More recently, mobility platforms such as the “unicorns” (i.e., $1B+ start-ups) *Uber*, *Bolt*, *Grab*, *GoJek*, *Didi Chuxing*, and *Careem* aim to integrate a variety of different services, making them more multifaceted and convenient for users. Initially focusing on ridesharing with a better matchmaking than conventional taxis, they now highlight the value proposition of multifunctionality, with ridesharing being just one of many features [5]. For instance, while *Grab* positions itself as the “Everyday Everything App”^1^, *Careem* claims to be a “hassle-free, one stop solution for […] daily needs”^2^.

Despite the massive attention that super apps receive in the media landscape and gray literature (e.g., Refs. [[6], [7], [8]]), and the size and market dominance they (are aiming to) reach, the scientific literature has largely overlooked the phenomenon of super apps so far.^3^ Previous publications have mainly examined the emergence of Asian super apps including *WeChat* ([[9], [10], [11]]) and *LINE* [12] (see also [2]). These studies from the communication literature make important contributions in providing an initial overview of the worldwide impact of super apps on social, cultural, and political dynamics ([2,9,11]) as well as the reasons for using super apps and usage behavior ([10,12]).

However, to the best of the author's knowledge, there are no studies so far in the business and management literature that address super apps. This leads to a lack of understanding of the super app phenomenon from a firm perspective, including the question why some digital platforms follow a super app strategy and others do not. Although platforms' ability to integrate multiple services and create multi-platforms is well described in previous studies (e.g., Refs. [4,[13], [14], [15]]), this literature does not fully capture the scale of super apps, which aim to offer various services under one brand and cover multiple aspects of daily life, all accessible through one app. Also, studies with a cross-sectoral perspective and empirical evidence from areas other than social media and communication are lacking.

The present study aims to fill this knowledge gap. It offers preliminary insights into the growth motivation of digital platforms, culminating in the integration of non-related services and the pursuit of a super app strategy. To this end, a quantitative analysis of data from 380 digital platforms in the mobility sector is conducted, followed by a case study to gain deeper insights.

The analyses address the following questions.

RQ1 Which digital platforms follow a super app strategy and what factors determine their success?

RQ2 Why do digital platforms aim for a super app status and how do they reach it?

By providing detailed insights into the evolution of digital platforms from single-purpose to multi-purpose apps, the article contributes to the literature on the platform economy ([3,16,17]), and in particular platforms’ strategic decision making ([14,18,19]) and platform competition ([20,21]). Due to the focus on ridesharing platforms, there is also a sound contribution to the transportation literature, particularly on platformization and integration in urban transport ([22,23]). The estimated regression model provides a better understanding of the enabling factors for the pursuit of a super app strategy. The case study further reveals the motivations to become a super app, and the incremental steps that are involved. Finally, a conceptual model and testable propositions are forwarded to stimulate additional studies on this timely, largely unresearched subject.

The remainder of the article proceeds as follows. The next section reviews the relevant literature. In Section 3, methods and data for the quantitative and qualitative analyses are described. Section 4 contains the results and Section 5 the discussion. Finally, the article ends with concluding remarks including some lines for future research.

## 2 Literature review

Digital platforms that connect different kinds of users have become increasingly popular in recent years and many areas of everyday life would be inconceivable without them. As a result, platform providers are encountering new growth opportunities, while traditional business rules have been radically changed [24]. Indeed, despite some inhibitors such as the lack of technological infrastructure, lack of complementary asset providers, and unconducive local regulations, the most successful platforms have experienced unprecedented growth [16]. Network effects play an important role in enabling such growth. The more users a platform has on both sides of the multi-sided market it creates, the more all users benefit from each other [25]. This makes the platform more interesting for additional users to join. However, in order to benefit from network effects in the first place, platforms need to reach a certain size (the critical inflection point), which exposes the ‘chicken-and-egg dilemma’ that emerging digital platforms face [17]. Other factors that are associated with the rapid growth of digital platforms are the “asset-light” business model and the intangible product of matchmaking [26]. According to Gawer [18], the scope of digital platforms is so narrow that it excludes core assets and most workers. The core business of digital platforms is enabling and supporting transactions between previously unmatched demand-side and supply-side participants [3]. One of the main sources of revenue for digital platforms are therefore transaction fees, which are paid by users at a small fraction of the actual price of the products or services. Taken together, the above considerations thus illustrate that digital platforms have the ability to grow rapidly, but also that growth (i.e., more users, more transactions) is a constant and compelling requirement of the platform business model.

### 2.1 Digital platforms’ growth strategies

Digital platforms use different strategies to accomplish growth. In the following subsections, four common growth strategies based on Ansoff [27] are described, which differ in terms of the time and resources they require and the risks involved. Although they represent distinct paths, Ansoff ([27], p. 114) notes that “in most actual situations a business would follow several of these paths at the same time.”

#### 2.1.1 Market penetration

A market penetration strategy describes the process of bringing products to an existing market in which the same or similar products are already available, with the aim of capturing market share from competing firms. From inception, many digital platforms focused on a specific target group or market segment [15]. Due to better access and efficiency (e.g., through the use of smartphones) and by creating low-end markets or leveraging excess capacity, they quickly contested market share from incumbent competitors [28]. According to Knee [29], this is critical because successful platforms need a minimum market share at which the network can achieve financial breakeven. To further support rapid market penetration, digital platforms also often tend to conflict with existing legal frameworks and exploit legal gray areas ([30,31]).

#### 2.1.2 Market development

Market development strategies are used to identify and develop new opportunities for selling products and services in previously unexplored markets. In the case of digital platforms, this refers in particular to new geographical markets ([32,33]). Unlike traditional firms, the internationalization of digital platforms is not a lengthy product of a series of incremental decisions; rather, they are able to internationalize rapidly due to network effects as well as the flexible and highly scalable platform business model [34]. Platforms can diffuse even quicker if they can serve homogeneous user needs across different regions and little adaptation of the business model is needed [32]. Stallkamp and Schotter [33] further found that digital platforms that are able to generate cross-country network effects are more likely to expand internationally compared to those whose network effects extend only to national markets. Ojala et al. [35] summarize four phases in the internationalization process of platforms – establishment, early internationalization, commercialization, and globalization. The study also argues that platform internationalization is resource dependent and that networking with actors controlling such resources in the target market is necessary [35].

#### 2.1.3 Product development

The product development strategy involves introducing products with novel and distinctive characteristics into an existing market. In the case of digital platforms, this can extend to both complementary or substitute products and services. Consider that users might find a hotel booking platform more useful if it also includes offers for private accommodations and if the stay can be combined with leisure activities and tour packages. This way, platforms can benefit from *indirect* network effects [36] and are more likely to reach critical mass [37].

#### 2.1.4 Diversification

Diversification is usually seen as the growth strategy that involves the highest risk, as it requires new skills and new techniques [27]. For digital platforms, this holds true to a lesser degree when compared to traditional firms (that focus on physical products). For example, on the technical side, it is easier for digital platforms to launch services in upstream or downstream markets, or even in markets that are not in close proximity. Simply put, there is not much difference in matching different users, be it for food, grocery, or parcel deliveries, or for ridesharing services. In the literature, platforms' integration of new services is referred to as platform envelopment. It describes the “entry by one platform provider into another's market by bundling its own platform's functionality with that of the target's so as to leverage shared user relationships and common components” ([14], p. 1271). According to Staykova and Damsgaard [38], this is essential for platforms as they need to ensure constant evolvability to remain competitive and achieve lock-in effects.

### 2.2 Super app strategy

Adopting a super app strategy is closely linked to diversification, as it involves entering (multiple) new markets with (multiple) new products or services. Schreieck et al. [4] calls this an assemblage strategy, a full integration of digital platforms of different types. From a platform economics perspective, whether or not platforms pursue such strategy is part of their boundary decision regarding the platform sides. It is a strategic decision that concerns “the configuration (i.e., number of sides) and […] how the sides that are associated to the platform are composed” ([18], p. 2). Accordingly, creating a super app denotes the strategic decision of increasing the number of sides of the platform, which can create benefits but also risks. In making such strategic decisions, platforms base their choices on immediate circumstances and their available internal resources [19].

Internal resources encompass both tangible and intangible assets, capabilities, and capacities that the platform firm possesses (Table 1). They are integral to its ability to create, deliver, and capture value within its ecosystem [39].

**Table 1:** Digital platforms’ internal resources.

Num.	Internal resource factor	Definitions
1	Human capital	The combined skills, knowledge, and capabilities of a firm's employees that contribute to its operational efficiency and value creation.
2	Organizational capital	Knowledge and experience that is institutionalized and codified, and utilized through databases, patents, processes, etc.
3	Technological infrastructure	The integrated network of hardware, software, and digital systems that underpin a firm's operations, supporting its internal processes and enabling innovation.
4	Financial resources and performance	The capital, funding, and monetary assets available to a firm, as well as its ability to effectively manage and generate returns from these resources.
5	User base	The aggregate number of individuals or entities actively engaging with the firm's services or content.

A crucial internal resource is human capital, demonstrated by previous research linking it to firm performance and strategy ([40,41]). Particularly for a super app strategy, skilled employees are essential for developing and seamlessly integrating new features, services, and functionalities. Furthermore, the firm's capacity to innovate hinges on organizational capital such as patents, trademarks, and copyrights ([42,43]). Ahmed et al. [44] suggest that human and organizational capital collectively contribute to a platform firm's agility within a rapidly shifting environment. Technological infrastructure likely also plays a major role [43]. A robust and scalable technological foundation that can accommodate a wide range of services and functionalities seamlessly is needed for a super app, especially in the fields of cloud computing, in-memory databases, and analytical solutions for big data [45]. Prior research within the domain of technology-based firms underscores that successful transformations often depend on the accumulation of financial resources and strategic partnerships ([46,47]). Notably, for the execution of a super app strategy, financial stability is requisite to underpin the development, maintenance, and expansion of the platform's multifaceted offerings. This stability can be gauged both by the nature of funding and the total funding amount raised. Additionally, Wang and Li [48] demonstrate that digital platforms' relevant investments exert significant influence on firm performance. Finally, in the context of digital platforms, the user base stands as a critical resource [33]. An active and engaged user base offers a network advantage, cross-promotion possibilities, and the potential to encourage the adoption of new services within the super app framework.

## 3 Methodology

To gain comprehensive insights into this topic, I am employing a mixed methods approach that combines quantitative and qualitative analyses [49]. The study is explanatory in nature, which means that the qualitative data help explain or build upon initial quantitative results [50]. The qualitative component complements the quantitative analysis by providing a richer understanding of the underlying motivations and contextual factors influencing digital platforms' decisions to integrate non-related services and pursue a super app strategy. It is important to note that due to the relative novelty of the super app phenomenon and the paucity of (longitudinal) data, this combination allows for a more holistic and nuanced exploration of the topic.

### 3.1 Quantitative analysis

#### 3.1.1 Variables and data

For the quantitative analysis, I utilize data obtained in December 2022 from *Crunchbase*, a database provider and news portal for corporate and business information, with a focus on technology companies and investors. Using the *Crunchbase* query builder, organizations that are either listed in the category “ride sharing” or in the category “app” in combination with the keywords “ride sharing”, “ride hailing”, “ride sourcing”, or “carpooling”^4^ are filtered. I also hand searched for additional relevant organizations that might not have been captured by the previously mentioned search criteria. Given that potential super apps typically require a certain scale, the results were restricted to organizations that are listed as active, were founded before 2020, and have more than 50 employees. This process yielded a total of 603 records. After removing duplicates and irrelevant organizations (e.g., those solely providing technology without operating a ridesharing platform), 380 records remain.

The data set includes 22 variables (Table 2). The dependent variable is the *SuperApp* status (D01), coded as a binary response, where “1” indicates that the organization is a super app. This information was added manually by checking whether, in addition to mobility-related services, the platforms also provided at least two other services. This was the case for 16 platforms (Table 3).

**Table 2:** Study variables and descriptives of the sample (n = 380).

Variable		Description	Category	Observations (% of sample)	Min.	Max.	Mean (SD)
H01	NoEmployees	Total no. of employees	51–100	130 (34.2)	–	–	–
			101–250	107 (28.2)	–	–	–
			251–500	53 (13.9)	–	–	–
			501-1000	34 (8.9)	–	–	–
			1001–5000	33 (8.7)	–	–	–
			5001–10,000	9 (2.4)	–	–	–
			10,001+	14 (3.7)	–	–	–
O01	NoTrademarksReg	Total no. of registered trademarks	–	–	0	189	2.79 (13.42)
O02	NoPatentsGranted	Total no. of patents granted	–	–	0	975	6.22 (57.14)
T01	NoActiveTech	Total no. of technologies in use	–	–	1	142	35.21 (28.98)
T02	NoApps	Total no. of apps	–	–	1	137	3.44 (9.71)
F01	TotFunding	Total founding amount raised in USD (log scale)	–	–	8.99	23.95	17.61 (1.87)
F02	TotEquityFunding	Total equity funding amount raised in USD (log scale)	–	–	8.99	23.64	17.29 (1.73)
F03	NoInvestors	Total no. of investors	–	–	1	116	6.07 (11.17)
F04	NoLeadInvestors	Total no. of lead investors	–	–	0	28	1.74 (3.25)
F05	NoFundingRounds	Total no. of funding rounds	–	–	0	34	2.86 (4.36)
F06	NoInvestments	Total no. of investments	–	–	0	30	0.38 (2.54)
F07	NoLeadInvestments	Total no. of lead investments	–	–	0	10	0.17 (1.05)
F08	NoAcquisitions	Total no. of acquisitions	–	–	0	29	0.77 (2.91)
F09	Acquired	Acquisition status (=1 if the organization was acquired)	–	57 (15.0)	–	–	–
F10	NoExits	Total no. of exists	–	–	0	7	0.07 (0.55)
U01	NoVisits	Total no. of website visits in the last month (log scale)	–	–	0	21.59	8.33 (3.56)
U02	WebTrafficRank	Global website traffic rank, as compared to all other websites on the web (log scale)	–	–	3.14	16.19	14.41 (2.09)
C01	Age	Platform age in years	–	–	4	24	9.13 (4.77)
C02	NoPortfolioOrg	Total no. of portfolio organizations	–	–	0	25	0.31 (1.97)
C03	NoProductsActive	Total no. of products active	–	–	1	91	12.27 (14.23)
C04	IPO	IPO status (=1 if public)	–	18 (5.0)	–	–	–
D01	SuperApp	Super app status (=1 if considered a super app)	–	16 (4.2)	–	–	–

**Table 3:** Overview of super apps in the mobility sector (n = 16).

Start-up	Founded	Head-quarters	Available in	Integrated services a					
				Ride-sharing	Food delivery	Parcel delivery	Grocery delivery	Payment	Other
Bolt	2013	Estonia	46 countries in Europe, Africa, Asia, and Latin America	●	●		●		e-scooter and car sharing
Cabu	2016	USA	USA, Nigeria	●	●	●	●	●	home cleaning, beauty and salon services, car wash, etc.
Careem	2012	UAE	12 countries in Africa and Asia	●	●	●	●	●	bike sharing, mobile bills and recharge service
Didi Chuxing	2012	China	16 countries in Europe, Africa, Asia, Latin America, and Oceania	●	●	●		●	several mobility (bus, bike sharing, business travel, freight, etc.) and financial services (loans, insurances)
Gett	2010	UK	10 countries in Europe and Asia	●		●			
Gojek	2009	Indonesia	Indonesia, Singapore, Vietnam	●	●	●	●	●	e-commerce, pharmacy, entertainment (movies, live events), financial services (loans, insurances, investments)
Gozem	2018	Togo	8 countries in Africa	●	●	●	●	●	
Grab	2012	Singapore	8 countries in Asia	●	●	●	●	●	financial services (insurances, investments)
Halan	2017	Egypt	Egypt, Ethiopia, Sudan	●				●	e-commerce, financial services (loans)
Hugo	2016	El Salvador	6 countries in Latin America and the Caribbean	●	●		●	●	pharmacy, entertainment, e-commerce, financial services, etc.
Ola	2010	India	India, Australia, New Zealand, UK	●				●	several mobility (car sharing, business travel, etc.) and financial services (loans, insurances)
Pathao	2015	Bangladesh	Bangladesh and Nepal	●	●	●		●	e-commerce, pharmacy, financial services (loans)
Pronto	2017	Mexico	Mexico	●	●		●		e-commerce, pharmacy
Safeboda	2015	Uganda	Uganda, Nigeria	●		●		●	mobile bills and recharge service, financial services (money transfer, bill payments)
Uber	2010	USA	approx. 72 countries in North America, Europe, Asia, Africa, Latin America, and Oceania	●	●	●		●	several mobility services (bike and e-scooter sharing), freight, financial services (debit account, debit card, digital wallet)
Yandex Go	2011	Russia	19 countries in Europe, Asia, Africa, and Latin America	●	●	●	●		

a Note that the availability of services may vary across different geographical markets.

The independent variables to explain the super app status reflect the internal resources: human capital (H01), organizational capital (O01-02), technological infrastructure (T01-02), financial resources and performance (F01-10), and user base (U01-02). In addition, some variables are included to control for the firm's age (C01), the number of portfolio organizations (C02), the number of active products (C03), and the IPO status (C04).

For *NoEmployees* (H01), the category “51–100” is used as the reference group, while the remaining are coded as dummy variables. *Acquired* (F09) and *IPO* (C04) are also dummy variables, and the remaining are continuous variables.

#### 3.1.2 Model estimation, analysis, and validation

The decision of pursuing a super app strategy can generally be expressed with the following regression (equation (1)):

(1) $$y_{i}^{*}=\beta_{i}\times x_{i}+\varepsilon_{i}$$

where $y_{i}^{*}$ is a latent variable representing the level of benefit firm i perceives from pursuing a super app strategy, $x_{i}$ a vector of explanatory variables, $\beta_{i}$ the regression coefficients, and $\varepsilon_{i}$ the model errors with normal distribution assumption. The level of benefit is not observable. What is observable is the binary variable y of the super app status, which can be explained by the following relationship (equation (2)):

(2) $$y=\left\{ \begin{array}{c} 1\;if\;y_{i}^{*}>0\\ 0\;otherwise \end{array}\right.$$

Due to the dichotomous nature of the dependent variable, a binary probit model can be estimated using the maximum likelihood method as follows (equation (3)):

(3) $$\ln\;L\left(\beta |x_{i},y_{i}\right)=\sum_{i=1}^{N}\left(y_{i}\;\ln\;\varphi \left(x_{i}\beta \right)+\left(1-y_{i}\right)\ln\left(1-\varphi \left(x_{i}\beta \right)\right)\right)$$

where the remaining unknown φ represents the standard normal cumulative distribution function.

Marginal effects (ME) are also computed to measure the change in value of the dependent variable through the change in value of a specific explanatory variable, while the other explanatory variables are kept fixed (equation (4)):

(4) $$\frac{\partial E\left[y_{i}|x_{i}\right]}{\delta x_{i}}=\varphi \left(y^{\prime }x_{i}\right)$$

R software is used to perform the analysis and validation. First, a full model with all independent variables is trained. To identify significant covariates and confounders in the model, a bidirectional stepwise approach is utilized, where variables are iteratively added and removed while minimizing the Akaike information criterion (AIC). To evaluate the quality of the final model, I perform several tests and report the following goodness of fit metrics: the Omnibus test of model coefficients, the log-likelihood value, the McFadden Pseudo R-squared, the AIC, the correct predictions as well as the positive and negative predictive values.

To validate the predictive performance of the model, stratified cross-validation is used. Given that only 16 observations are associated with the super app attribute, I adopt a leave-one-out approach to detect overfitting and identify any influential observations that may have a large impact on the model's performance. Specifically, the learning algorithm is applied once for each of the observations with the super app attribute, using all other observations as a training set and the selected observation as a validation set. The model evaluation scores are then compared to summarize the performance of the model on new data.

### 3.2 Qualitative analysis

The analysis of quantitative data sheds light on the “which” and “what” questions concerning super apps (see RQ1). Qualitative data is considered more appropriate for answering the “why” and “how” questions [52]. As Eisenhardt ([53], p. 542) puts it, “qualitative data often provide a good understanding of the dynamics underlying the relationship, that is, the “why” of what is happening”. Hence, to understand why digital platforms aim for a super app status and how they reach it (RQ2), I employ a case study approach [52] with a content analysis [54], which is a widely used method in qualitative research. Based on a purposeful sampling procedure, *Uber* is considered as the most instructive case for twofold reason. First, according to *Crunchbase* data, it is the largest mobility platform in terms of valuation ($82.4B), funding amount ($25.2B), and estimated revenue range ($10B), as well as the most popular in terms of monthly app downloads (18 M+) and website visits (89 M+). Second, the *Uber* case has been widely studied in the scientific literature both from institutional (e.g., Refs. [31,55]), organizational (e.g., Refs. [56,57]), and behavioral perspective (e.g., Refs. [26,58]), and there is wealth of available data from online sources (Table 4).

**Table 4:** Main online sources used in the case analysis.

Type	Source	URL
Press releases	Uber website	https://investor.uber.com/news-events/default.aspx
Financial reporting	Uber website	https://investor.uber.com/news-events/default.aspx
News blog	Uber website	https://www.uber.com/newsroom/news/
Social media	Uber Twitter account	https://twitter.com/Uber
Investment and funding information	Crunchbase	https://www.crunchbase.com/organization/uber
Newspaper	The Guardian	https://www.theguardian.com/news/2022/jul/10/uber-files-timeline-parisian-eureka-moment-global-domination
Newspaper	TheStreet	https://www.thestreet.com/technology/history-of-uber-15028611
Newspaper	Business Insider	https://www.businessinsider.com/ubers-history

In the content analysis, relevant excerpts and text paragraphs from the available materials were systematically coded to aggregate content. The codes for the respective growth strategies were predefined (i.e., market penetration, market development, product development, and diversification). Additionally, inductive codes were developed to capture information related to the motivations for starting and ending a strategy. The findings obtained from this coding scheme are then discussed against a backdrop of the available literature.

## 4 Results

### 4.1 Model results

Table 5 contains the model results including the average marginal effects. Eight independent variables are included in the final regression model, of which all are statistically significant (p < 0.10). Regarding the Omnibus test of model coefficients, the p-value (0.00) is below the critical value of 0.05. Hence, it can be concluded that the model specification is an improvement over the baseline model. The McFadden Pseudo R-squared corresponds to 0.54. Note, hereby, that values beyond 0.5 indicate an excellent fit [59]. The predictive accuracy of the model is 0.98. However, given the significant skewness in the data set and the presence of only 16 observations with the super app attribute (=1), it is of particular interest to assess whether the model is able to predict actual “1s”. While the negative predictive score is perfect at 1, the positive predictive score is 0.44, signifying that the model correctly identifies approximately 44% of observations with a “1” in the dependent variable. Overall, it can be concluded that the predictive performance of the model is satisfactory.

**Table 5:** Model summary.

Variable	Coefficient	Std. error	Ave. ME
(Constant)	2.1930	1.5880	
Age	−0.1539**	0.0720	−0.0065
NoPortfolioOrg	3.3778**	1.4177	0.1432
NoInvestments	−1.8619*	1.0077	−0.0789
NoLeadInvestments	−1.9431**	0.8964	−0.0824
NoExits	2.4571*	1.2790	0.1042
NoFundingRounds	0.2638***	0.0625	0.0112
TotEquityFunding	−0.2597**	0.1010	−0.0110
NoPatentsGranted	−0.0078**	0.0033	−0.0003
**Model summary statistics**			
Log likelihood:	−30.36894 (df = 9)		
AIC	78.73789		
McFadden Pseudo R-squared:	0.542220		
Correct predictions	0.9763158		
Positive predictive value	0.4375		
Negative predictive value	1		

Note: *p < .10; **p < .05; ***p < .01.

The model configuration also holds the stratified cross validation; the McFadden Pseudo R-squared remains robust in the validation sets (ranging between 0.5190 and 0.5502, mean = 0.5463). Based on these results, overfitting is unlikely to be a major issue. Additionally, there is no evidence of influential observations that would substantially affect the model's performance.

The interpretation of the coefficients of the independent variables follows below.

• *Age*: The negative coefficient indicates that rather young platforms have reached the super app status. On average, the platforms in the data set considered as super apps are 8.75 years old, compared to an average of 9.16 for the remaining platforms. This suggests that platforms that adopt a super app strategy have been able to achieve instant growth from inception. One possible explanation for this result is that younger platforms have a greater need to differentiate themselves from established competitors and gain market share quickly. In addition, they might be more agile and adaptable than older platforms, which can make it easier for them to pivot towards a super app strategy and integrate new services and features into their platform. In contrast, some of the more established platforms have either not yet attained the super app status or will not do so at all. For instance, this could be due to the lack of internal resources and capabilities, which could result in a higher likelihood of specialization in specific niche products or markets. Alternatively, they might have already solidified their brand identity and customer base, which makes it more difficult to shift to a super app strategy with a completely different value proposition [60]. The latter could also be the reason Elon Musk rebranded *Twitter* as *X* after announcing his super app (or “everything app”) ambitions [61].
• *NoPortfolioOrg*: Even though super apps operate as one brand to the outside world, their organizational structure is usually quite convoluted simply due to their size and operating in different geographic markets with heterogeneous products and services. It is therefore not surprising that the total number of portfolio organizations has a positive impact on the super app status. In addition, it might not always be the best option to fully integrate an auxiliary platform firm. Schreieck et al. [4] argues that the decision of full versus partial integration into the focal platform may hinge on the nature of network effects. Previous studies confirm that even in the presence of increased network effects, sometimes differentiation and operating two different platforms is more beneficial [62] and that the integration of new platforms can even have a negative impact on other platform services [63]. Moreover, having a larger number of portfolio organizations can provide the platform with a competitive advantage by enabling it to negotiate better deals with partners and suppliers. Notwithstanding, the number of acquisitions was not found to be significant. Taken together, this possibly indicates that the super app status is not only realized through the acquisition of competitors, but rather also depends on organic growth and in-licensing [43].
• *NoInvestments*, *NoLeadInvestments*, *NoExits*: Interestingly, it is a higher number of exits, in combination with a lower number of investments and lead investments that contribute to the super app status. One possible explanation is that platforms pursuing a super app strategy aim for diversification and tend to be more active in non-related markets. Their business practices can therefore be considered more volatile and risk-taking, which includes trial and error. This was also observed by Zeng et al. [64] in their longitudinal case study of *Tencent* – the developer of the *WeChat* super app. The study notes that the repeated addition and connection of platform assets through the discovery of new and different ecosystem resources enabled diverse and greater opportunities for a novel reconfiguration [64]. In contrast, a high number of investments combined with few exits could indicate a specialization strategy of firms without super app status.
• *NoFundingRounds*, *TotEquityFunding*: The positive coefficient of *NoFundingRounds* indicates that platforms that pursue a super app strategy are performing well in terms of attracting funding. They therefore likely have a strong and dedicated investor base, which can provide strategic guidance, networking opportunities, and other resources [65]. Less likely, however, is a high amount of equity funding, where investors receive shares in the venture in return for their investment and the platform thus has more pressure to achieve short-term financial goals (e.g., profitability) and meet the investors' expectations [66]. Instead, platforms adopting a super app strategy may choose to raise funds through alternative sources, such as debt financing, crowdfunding, grants, or secondary market transactions, which can offer greater autonomy and agility in decision-making.
• *NoPatentsGranted*: Although statistically significant, the total number of patents granted has a very low contribution to explain the super app status. Nevertheless, it is left in the model as a significant confounder.

### 4.2 Case study results

#### 4.2.1 Uber market penetration

*Uber* initially launched its ride-hailing platform in 2010 in the San Francisco Bay area. The service has been introduced as a faster and more convenient alternative to conventional taxis, which can be hailed via a mobile app. Once the service was successful enough, *Uber* sought to expand into other cities across the country [15], starting with New York City in May 2011. The subsequent roll-out in the US market is described in Berger et al. [56] and Hall et al. [57]. Both studies found that *Uber* largely entered cities in population rank order, suggesting that market size (i.e., both available drivers and passengers) is the most important factor in the entry decision. This supports the assumption that platforms require a sufficient number of users and aim to reach a certain size through fast market penetration. Accordingly, Hall et al. [57] cited *Uber* executives as aiming to cover as much of the nation as soon as possible. Indeed, despite legal battles, fierce opposition by taxi drivers, and a number of allegations against its business practices [67], *Uber* diffused rapidly and was already available in the fifty most populous metropolitan areas by 2015. In this context, the literature has identified convenience, low fares, and off-peak availability (i.e., late evenings and nighttime) on the passenger side [68] as well as more flexible work arrangements and expected surpluses on the driver side [69] as key drivers of user adoption.

#### 4.2.2 Uber market development

It also did not take long for *Uber* to turn its focus to the international markets and introduce the ride-hailing concept in other countries. The international expansion started with the launch in Paris, France in May 2011. Similarly to its home market, *Uber* diffused quickly in Europe, initially focusing on major metropolitan areas before consolidating smaller cities. At the same time, *Uber* quickly gained a foothold in the Global South, where populous urban areas (despite lower income levels) represent attractive markets for platform firms. According to Hasselwander et al. [32], *Uber*'s rapid international expansion was enabled due to its highly replicable and scalable business model. Indeed, ride-hailing is experiencing great popularity around the globe as a convenient urban travel alternative, especially in areas that lack high-quality public transit [70]. Nevertheless, although *Uber* has entered many markets as a first-mover [32], it has faced stiff competition from local start-ups (especially in developing countries) and thus has been unable to scale sufficiently on a number of occasions. Strict local regulations also made it difficult to establish ride-hailing in other mostly more developed countries (e.g., Germany, Denmark, South Korea) [32]. The growth potential through international expansion was therefore only possible to a limited extent, and at some point, *Uber* even withdrew completely from some regions (e.g., in China and Southeast Asia).

#### 4.2.3 Uber product development

Watanabe et al. [71] demonstrates that *Uber*'s product developments accelerated as its growth rate increased. Originally, *Uber*'s service with luxury cars was pricier than conventional taxis. However, in July 2012, *Uber* introduced the cheaper *UberX* service with lower-cost hybrid vehicles, and later drivers could even use their personal vehicles. Several similar product developments followed afterwards such as *UberXL* (larger vehicles for up to 6 passengers), *UberBLACK* (luxury black cars with black leather interiors), and *UberGo* (smaller, fuel-efficient vehicles). In addition, *UberPool* was announced in August 2014, allowing passengers to share a ride based on proximity. In April 2018, *Uber* acquired shared mobility provider *JUMP* and subsequently integrated shared bicycles and e-scooters into its platform. From that point onward, *Uber* offered competing services, aimed at the same objective of transportation from point A to point B, despite the potential risk of cannibalizing its core ridesharing business. Nevertheless, the tendency of mobility platforms to cater the entire urban mobility market is to be explained by growth and profits motivations [37]. Unsurprisingly, *Uber* continues to target other mobility services and, for example, entered into cooperative agreements with some transit authorities to integrate public transit services. On the one hand, additional services allow *Uber* to reach a larger target group and achieve lock-in effects. On the other hand, it is part of the natural spin-off dynamics of digital platforms that is driven by people's preferences shift, advancement of ICT, and paradigm change [71].

#### 4.2.4 Uber diversification

**Fig. 1:**
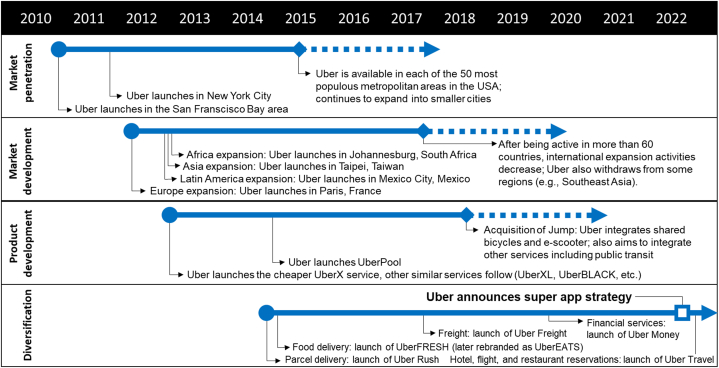
Uber case study: overview of growth strategies.

Diversification activities started in April 2014 with the launch of *Uber Rush*, a parcel delivery service, and *UberFRESH* (later rebranded as *UberEATS*), a food delivery service, in December 2014. These services were the first that did not involve the transportation of people, but represent (transportation) side horizontals. Consider here that besides leveraging the existing platform infrastructure, also the drivers can be the same as for the ridesharing services (so-called multihoming). The potential synergies and increased network effects are thus evident, although Chung et al. [63] observed some cannibalization effects for the core business in a case study of *UberEATS* in New York City. In October 2019, *Uber* diversified vertically into financial services with the launch of *Uber Money*. It gives drivers instant access to their earnings through debit accounts. Users also have access to a wallet where earning and spending histories can be tracked. Another feature, *Uber Travel*, allows users to organize hotel, flight and restaurant reservations. To benefit from demand spillovers [72] and extended lock-in effects, the integration of similar complementary services can be expected in the future. Consequently, *Uber* officially announced its super app strategy in April 2022 (Fig. 1).

## 5 Discussion

### 5.1 Conceptual model and propositions

Digital platforms are a special type of firm that have emerged in a variety of domains in recent years, in many cases challenging incumbent market participants and disrupting entire industries. One of their distinctive features is the ability to diffuse quickly and achieve rapid growth, and integrate various products and services, culminating in the emergence of super apps. The above analyses aimed at a better understanding of this phenomenon.

**Fig. 2:**
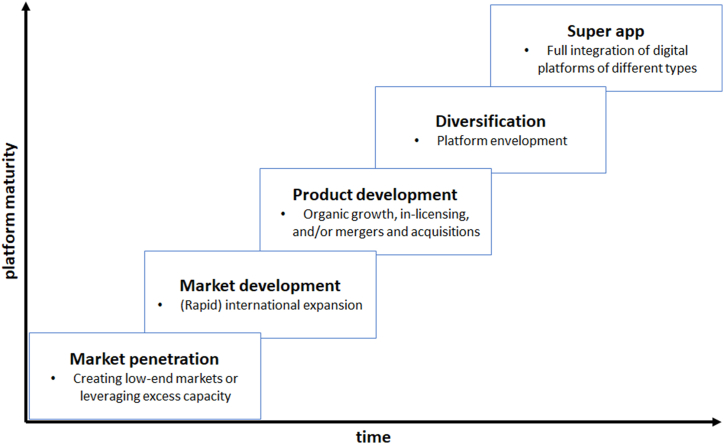
Conceptual model of digital platforms' growth strategies: the path towards a super app.

Indeed, it has been found that one of the main goals of digital platforms is to achieve growth, which often takes precedence over other goals such as profitability. Due to the “asset-light” business model and the nature of their intangible products, platforms need to grow to create value. It is therefore imperative that they constantly attract and retain additional users on each side of their multi-sided market and that these users complete as many transactions as possible. For this purpose, digital platforms follow different growth strategies as illustrated in Fig. 2.

Typically, digital platforms start with a market penetration strategy from inception. By undercutting competitors’ prices or leveraging excess capacities, digital platforms aim to quickly gain market share. Similar to “international new ventures” and “born global” firms [73], digital platforms have the capability to internationalize at an early stage, which corresponds to the second growth strategy, market development. Digital platforms often start international expansion activities in markets in close proximity (e.g., geographic or cultural proximity), but ultimately target a global expansion [74]. In the next phase, a product development strategy aims to achieve growth by adding additional products and services to the platform to attract previously unserved segments in the same market. This can be achieved through organic growth, in-licensing, and/or by acquiring competing firms. In a similar way, digital platforms then usually diversify into vertical markets with unrelated products and services by combining functionality with competing platforms (platform envelopment). Once digital platforms have gone through all four growth strategies – with the individual phases usually overlapping – they are likely to turn into super apps, and fully integrate multiple digital platforms into a single solution, accessible through a single app.

Nevertheless, by far not all digital platforms reach the final stage of growth strategies, equivalent to the status of a super app. The lack of financial resources, the lack of knowledge, infrastructure or other internal resources, as well as strong competition can inhibit growth. Instead, the regression analysis shows that it is rather young, risk-taking firms that – backed with enormous funding – are able to achieve rapid growth from the outset and quickly move to the next possible growth strategy to ensure constant growth.

Based on the above findings, the following testable propositions are put forward.

P1 *Digital platforms that are able to achieve growth through market penetration, market development, product development, and diversification, will eventually pursue a super app strategy. Hence, digital platforms are not born “super”. Instead, attaining the super app status is an incremental process based on the successful exploitation of various growth strategies.*

P2 *: Young and agile digital platforms that prioritize innovation and risk-taking in their decision-making are more likely to follow a super app strategy. Their approach can lead to more volatile outcomes, but also allows for faster adaptation to changing market conditions and user needs.*

### 5.2 Managerial implications

The findings from this study hold several important managerial implications for digital platforms. First, it highlights that becoming a super app is not an innate characteristic of digital platforms but rather an outcome of a series of strategic decisions. Therefore, digital platforms should engage in long-term planning and carefully assess their potential to adopt a super app strategy, taking into account their available internal resources [39], innovation and change capabilities [75], and competitive landscape [76]. Long-term planning includes foresight practices such as scenario planning to anticipate uncertain futures [77]. This facilitates a realistic assessment of whether digital platforms have the prerequisites and resources to pursue a super app strategy and whether they should actively pursue it.

Second, managers must recognize that becoming a super app involves a series of distinct growth strategies, each building upon the foundation of the previous one. These strategies represent critical steps in the evolution towards the super app status. Importantly, none of these steps can be skipped or expedited without careful consideration of their outcomes. Moreover, it is essential to understand that certain valuable resources and capabilities are cultivated during earlier stages of growth. For instance, the user base developed during the market penetration phase serves as a crucial asset during subsequent phases. This underscores the interconnected nature of these growth strategies and the need for strategic alignment across all stages. Managers should approach each step methodically, leveraging the internal resources and knowledge accumulated along the way to successfully progress towards the goal of becoming a super app.

Third, the insights gleaned from these growth strategies not only inform a digital platform's own journey towards becoming a super app but also provide valuable guidance for recognizing the potential trajectories of competitors. By closely analyzing the development stages and strategic decisions of competitors [75], digital platforms can gain a clearer understanding of which of them are likely to evolve into super apps themselves. This understanding is crucial for proactive strategic adjustments. Platform firms can adapt their own strategies accordingly, which may involve specialization in niche markets or focusing on distinctive offerings to differentiate themselves in a competitive landscape. Recognizing the potential super app competitors early allows digital platforms to make informed decisions about resource allocation, partnerships, and market positioning to maintain competitive advantages.

## 6 Conclusion and future research directions

This study has analyzed which digital platforms are turning into super apps, and why and how they are evolving from single-purpose to multi-purpose apps. It provides preliminary insights on the incremental steps on the path towards a super app and the motivating role of growth objectives. The regression model further reveals the factors that determine whether a platform is capable of pursuing a super app strategy. In addition, the results of this study are enhanced by a conceptual model and testable propositions, as well as a discussion of managerial implications.

Future research could address the limitations of the methods and data used in this study. For instance, the study sample comprises only 16 platforms with the super app attribute. While this limitation has been addressed through the use of cross-validation techniques, expanding the analysis to include super apps outside the mobility sector could contribute to either confirming or challenging the findings of this study. While this study has primarily emphasized internal resources as predictors of the super app status, it is important to note that it does not assert that these factors are the sole determinants. The significant concentration of super apps in East and Southeast Asia suggests that attaining the super app status also depends on local regulation and user preferences. There is likely also a measurement bias due to the paucity of available data from digital platforms. Human capital, for instance, is solely measured by the total number of employees, which fails to capture differences in relevant skills and expertise at the management level. Lastly, more profound insights can be derived from the analysis of longitudinal or panel data. Considering the changes in variables of interest over time allows for a more comprehensive understanding of the evolution of digital platforms into super apps.

Other promising lines of research revolve around the following.

i. **Performance and competition**: Although this study sheds some light on the motivations and reasons why digital platforms develop into super apps, additional studies are needed to better understand how the super app strategy influences their business policies and practices. For example, empirical studies are needed to determine external network effects and lock-in effects of integrating complementary or unrelated services. Furthermore, the emergence of super apps is transforming the competitive landscape by breaking down industry boundaries and creating new opportunities for platform providers. For example, consider that messaging platforms like *KakaoTalk* now offer ridesharing services, while ridesharing platforms like *Uber* offer food delivery and other services. These changes are creating a more fluid and competitive market, where platforms across different industries are competing with each other. In this new landscape, platform providers must integrate new services not only to achieve growth and leverage its users into new markets, but also to counteract the network effects of a rival platform and to underpin their position in cross-industry competitions.
ii. **User acceptance and usage behavior**: There is also not yet a clear understanding of the super app users and their characteristics. Potential users are de facto limited to smartphone users, but it is likely that not all of them appreciate the idea of obtaining all kinds of services through a single app. Frameworks to study the acceptance of (technical) innovations (e.g., TAM or UTAUT model) can help identify potential adopters and the underlying reasons to use super apps [78]. Stated choice experiments can further provide insights on how many and which services they demand for. Subsequently, it is of interest to understand if and how the availability of super apps changes usage behavior. For example, is it likely that users will exhaust the entire portfolio of the super app and access services that they would not have used otherwise? Does the integration of new services have a positive effect on the use of other complementary services in the same app? And, are super app users likely to discontinue using single-purpose apps or do they continue using single-purpose apps at the same time (so-called multihoming)?
iii. **Regulation**: Furthermore, the emergence of super apps only adds to the existing controversies regarding digital platforms' ability to dominate markets, undermine competition, and the accusation that they tend to bypass tax and insurance obligations as well as concerns regarding user data privacy. Especially in Europe – where super apps are not yet very prevalent – the pressing question remains how digital platforms should interact with the external political forces [79]. In future research, scholars should thus analyze different regulatory approaches that governments and regulatory bodies could adopt to address the aforementioned concerns, as well as the potential impact this may have on entrepreneurship and business model development [80].

## Data availability statement

Data will be made available on request.

## CRediT authorship contribution statement

**Marc Hasselwander:** Writing – review & editing, Writing – original draft, Visualization, Validation, Software, Resources, Project administration, Methodology, Investigation, Formal analysis, Data curation, Conceptualization.

## Declaration of competing interest

The author declares that he has no known competing financial interests or personal relationships that could have appeared to influence the work reported in this paper.

## Footnotes

1 https://www.grab.com/sg/.

2 https://www.careem.com/.

3 As of August 13, 2023, the Web of Science database only contains 5 records (of which only one is a journal article) that include the term “super app(s)” in the title, abstract, or keywords.

4 Note that ridesharing is a concept that connects drivers and passengers on a platform. Ride hailing, ride sourcing, and carpooling are different types of ridesharing services. For a detailed presentation on this topic, the reader is referred to Shaheen and Chan [51].
